# Streamlining atrial fibrillation ablation management using a digitization solution

**DOI:** 10.1093/ehjdh/ztae041

**Published:** 2024-05-23

**Authors:** Jim O’Brien, Sergio Valsecchi, Fionnuala Seaver, Lorena Rosalejos, Diana Arellano, Kristine Laurilla, Gael Jauvert, Noel Fitzpatrick, Tamas Tahin, Ted Keelan, Joseph Galvin, Gabor Szeplaki

**Affiliations:** Atrial Fibrillation Institute, Mater Private Hospital, 71 Eccles Street, Dublin 7, D07 T92C, Ireland; Boston Scientific Inc., Natick, MA, USA; Atrial Fibrillation Institute, Mater Private Hospital, 71 Eccles Street, Dublin 7, D07 T92C, Ireland; Atrial Fibrillation Institute, Mater Private Hospital, 71 Eccles Street, Dublin 7, D07 T92C, Ireland; Atrial Fibrillation Institute, Mater Private Hospital, 71 Eccles Street, Dublin 7, D07 T92C, Ireland; Atrial Fibrillation Institute, Mater Private Hospital, 71 Eccles Street, Dublin 7, D07 T92C, Ireland; Atrial Fibrillation Institute, Mater Private Hospital, 71 Eccles Street, Dublin 7, D07 T92C, Ireland; Atrial Fibrillation Institute, Mater Private Hospital, 71 Eccles Street, Dublin 7, D07 T92C, Ireland; Atrial Fibrillation Institute, Mater Private Hospital, 71 Eccles Street, Dublin 7, D07 T92C, Ireland; Department of Cardiology, Zala Varmegyei Szent Rafael Hospital, Zalaegerszeg, Hungary; Atrial Fibrillation Institute, Mater Private Hospital, 71 Eccles Street, Dublin 7, D07 T92C, Ireland; Department of Cardiology, Mater Misericordiae University Hospital, Dublin, Ireland; Atrial Fibrillation Institute, Mater Private Hospital, 71 Eccles Street, Dublin 7, D07 T92C, Ireland; Department of Cardiology, Mater Misericordiae University Hospital, Dublin, Ireland; Health Sciences Centre, UCD School of Medicine, University College Dublin, Dublin, Ireland; Atrial Fibrillation Institute, Mater Private Hospital, 71 Eccles Street, Dublin 7, D07 T92C, Ireland; Department of Medicine, Royal College of Surgeons in Ireland, 123 Saint Stephen's Green, Dublin 2, D02 YN77, Ireland

**Keywords:** Atrial fibrillation, Catheter ablation, Outpatient care, Patient education, Knowledge, Patient engagement, Care digitalization

## Abstract

**Aims:**

Catheter ablation is a widely accepted intervention for atrial fibrillation (AF) management. Prior to undertaking this procedure, thorough patient education on its efficacy and potential complications is crucial. Additionally, educating patients about stroke risk management and anticoagulant therapy is imperative. At Mater Private Hospital in Dublin, we implemented a solution, integrating a customized treatment pathway and a mobile application. This patient-centred approach aims to optimize the clinical management of AF catheter ablation candidates, focusing on knowledge gaps and adherence to guideline-based care to enhance overall outcomes.

**Methods and results:**

The application automates pre-operative assessments and post-operative support, facilitating seamless patient–clinician communication. During the observation period (September 2022–April 2023), 63 patients installed the app. Patient adherence to the pathway was strong, with 98% of patients actively engaging in the treatment pathway and with 81% completing all pre-operative tasks. The average enrolment-to-admission duration was 14 days, and post-ablation tasks were fulfilled by 62% of patients within an average of 36 days. Operators perceived the solution as user-friendly and effective in enhancing patient connectivity. Patient satisfaction was high, and knowledge about AF improved notably through the solution, particularly concerning the recognition of symptoms and anticoagulation therapy-related complications.

**Conclusion:**

Our findings demonstrate the successful implementation of the app-based Ablation Solution, showcasing widespread patient use, improved adherence, and enhanced understanding of AF and its treatments. The system effectively connects healthcare providers with patients, offering a promising approach to streamline AF catheter ablation management and improve patient outcomes.

## Graphical Abstract

**Graphical Abstract:**
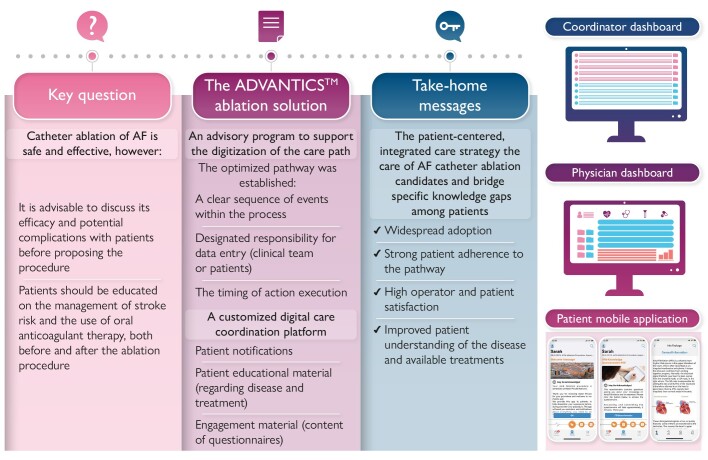

## Introduction

Atrial fibrillation (AF) stands as a prevalent cardiac rhythm disorder. Presently, it is estimated to affect ∼2–4% of adults, with an anticipated 2.3-fold increase attributed to extended life expectancy in the general population and intensified efforts to detect undiagnosed AF.^1^ The complexity of AF necessitates a comprehensive and multidisciplinary approach to patient management, with active patient involvement in collaboration with healthcare professionals. Existing guidelines underscore the significance of a coordinated, patient-tailored care pathway to deliver optimized treatment for AF patients.^1^ Patient awareness regarding AF and its management often remains limited, especially at the time of initial diagnosis when many treatment decisions are deliberated and made.^2–6^ It is imperative to ensure that patients receive appropriate information on treatment choices, adherence guidelines, potential consequences of non-adherence, and the realistic expectations of treatment outcomes to promote adherence effectively.

Catheter ablation of AF is a well-established intervention for preventing AF recurrences.^1^ While catheter ablation offers a safe and superior alternative to antiarrhythmic drugs for maintaining sinus rhythm and alleviating symptoms, it is advisable to discuss its efficacy and potential complications with patients before proposing the procedure. Furthermore, patients should be thoroughly informed about the clinical signs and symptoms of rare but potentially serious ablation-related complications that may manifest after hospital discharge. Additionally, patients should be educated on the management of stroke risk and the use of oral anticoagulant therapy, both before and after the ablation procedure.

In the Mater Private Hospital in Dublin, we have contributed to the development of a solution that encompasses a customized treatment pathway, the ADVANTICS™ Ablation Solution (Boston Scientific Inc., Natick, MA, USA), which incorporates a patient mobile application. This innovative approach is aimed at optimizing clinical practice in the management of patients undergoing AF catheter ablation. The project focuses on creating a patient-centred, integrated care strategy designed to streamline the care of AF catheter ablation candidates and bridge specific knowledge gaps among patients. The ultimate goal is to facilitate the implementation of guideline-based AF management and enhance patient outcomes.

This report seeks to provide an overview of the initial experiences with the implementation of the ADVANTICS™ Ablation Solution in clinical practice.

## Methods

### Solution description

The solution introduces changes to the traditional surgical pathway by standardizing, optimizing, and automating labour-intensive, paper-based procedures. Its primary goal is to support hospitals in improving waiting list management, streamlining operations, and enhancing the overall experience for healthcare providers and patients. The solution includes an advisory programme to support the digitization and a digital care coordination platform.

### Advisory programme on patient engagement digitization

Following initial interviews with all healthcare professionals involved in AF treatment at the centre, the patient journey at Mater Private was mapped, focusing on the communication steps and educational materials used. Based on the assessment, the pathway was then optimized and dedicated patient engagement materials were developed to be leveraged in the digital care path.

**Figure 1:**
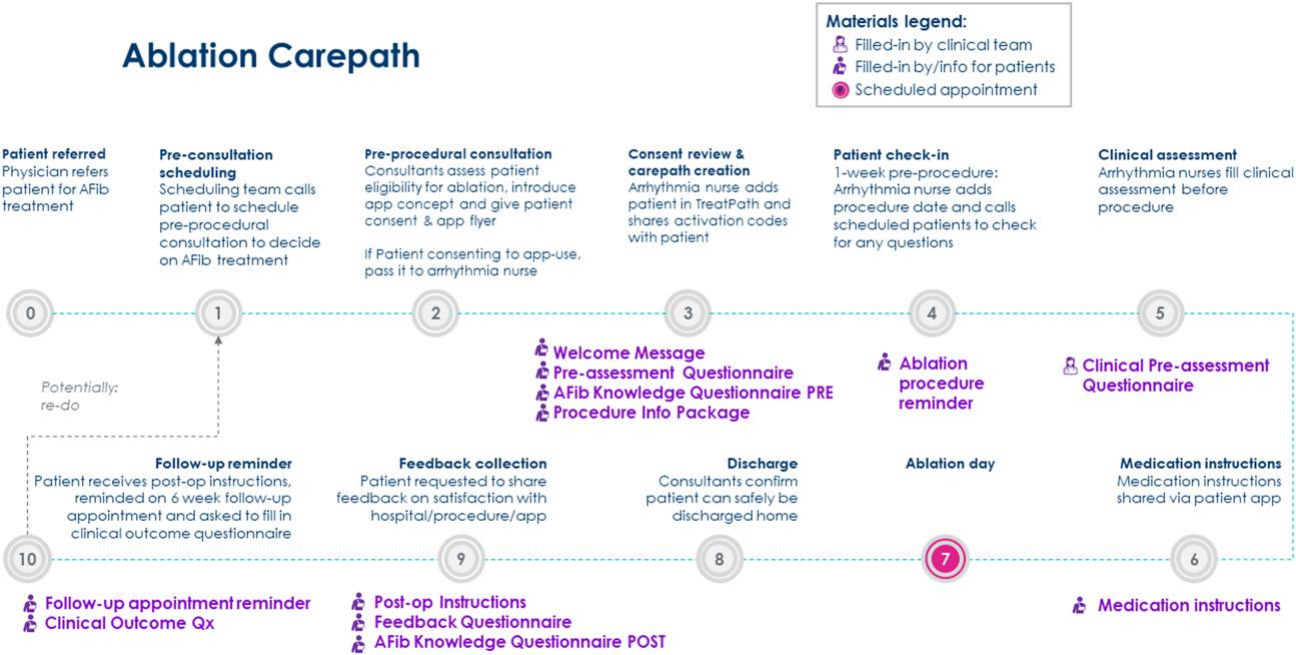
Description of the customized treatment path based on the application.

The platform underwent adaptation to align with the specific requirements and the optimized pathway related to the pre- and post-operative management of patients undergoing AF catheter ablation. This involved establishing a clear sequence of events within the process, defining the content of questionnaires, designating responsibility for data entry (whether by the clinical team or patients), determining the timing of action execution, specifying patient notifications, and shaping the educational material provided to patients regarding the disease and treatment (*Figure 1*). It was therefore established that the presentation of the digital care path and the mobile application to the patient would be conducted by the administrative staff during the pre-procedural consultation visit. Nurses would be responsible for clinical assessment, providing medication instructions via the application, and post-operative instructions. The clinical and feedback questionnaires would be completed by patients independently via the application, prompted by specific automatic reminders. The information material included information about the disease, consequences of AF, treatment options, role of anticoagulation, description of catheter ablation procedure (with risks), importance of lifestyle and risk factor modification. Upon the completion of the system setup, the staff received training on how to effectively utilize the web-based dashboard and patient materials were produced and distributed to facilitate the adoption of the application.

### The digital platform

The digital care coordination platform (Buddy Healthcare, Helsinki, Finland) comprises a user-friendly application for patients and a web-based dashboard for clinicians. The patient application offers the capability for patients to complete electronic forms, submit data from remote care devices, and engage in seamless communication with their clinical teams (see Supplementary material online, *Figure S1*). On the other hand, the clinician dashboard equips healthcare professionals with tools to enter data (see Supplementary material online, *Table S1*) and review patient information, respond to alerts, and foster efficient communication with patients (see Supplementary material online, *Figure S2*). This platform digitalizes the pre-operative assessment process and facilitates digital post-operative support, encompassing the automated collection and visualization of patient-reported experience and outcome measures. The system’s primary objective is to maintain effective communication between hospitals and patients who are awaiting appointments and surgeries. It offers the functionality to confirm a patient’s readiness for surgery, reduce the necessity for traditional outpatient visits and phone calls, enhance patient education and engagement, and streamline data capture.

### Evaluation design

Throughout the pilot the usage by patients and clinical team was closely monitored to ensure clinical team and patient satisfaction. The pilot evaluation of this solution commenced in September 2022. Every patient referred to the centre for *de novo* AF ablation underwent a pre-procedural consultation to assess the suitability of AF treatment. After verification of the availability of a mobile device by the patient or a caregiver, eligible patients for ablation were informed about the project. The ones who were interested were enrolled and invited to download the application, subsequently following the provided guidance. In April 2023, the operators were requested to complete a specialized questionnaire, evaluating their experience with the Ablation Solution. Patient feedback on their experience with the application was collected following their hospital discharge. The compliance of patients with the treatment pathway steps and their adherence to specified execution timings were closely monitored. Additionally, patients were given a dedicated questionnaire to assess their baseline knowledge of AF at the time of enrolment, with a follow-up assessment conducted after hospital discharge (see Supplementary material online, *Table S2*).

For the purpose of this analysis, all data were de-identified in compliance with European regulations (European General Data Protection Regulation—UE 2016/679) to safeguard personal health information. The data protection officer confirmed adherence to the relevant regulations. Since this project involved a retrospective analysis of prospectively collected clinical data in real-world settings, it was exempt from review and approval by the institutional review board. Patients had granted written approval to contribute data at the time of inclusion.

### Statistical analysis

Descriptive statistics are reported as means ± standard deviation for normally distributed continuous variables, or medians with ranges in the case of skewed distribution. Categorical data were expressed as percentages. Differences between mean data were compared by a *t*-test for Gaussian variables. Differences in proportions were compared by a χ^2^ analysis. A *P* value < 0.05 was considered significant for all tests. The results of the evaluation of the knowledge of AF conducted at the time of enrolment and after hospital discharge were compared by assessing the difference in the proportion of patients who answered the question correctly for single-choice questions and the patient-based average of correct answers for multiple-choice questions. All statistical analyses were performed by means of R: a language and environment for statistical computing (R Foundation for Statistical Computing, Vienna, Austria).

## Results

**Figure 2:**
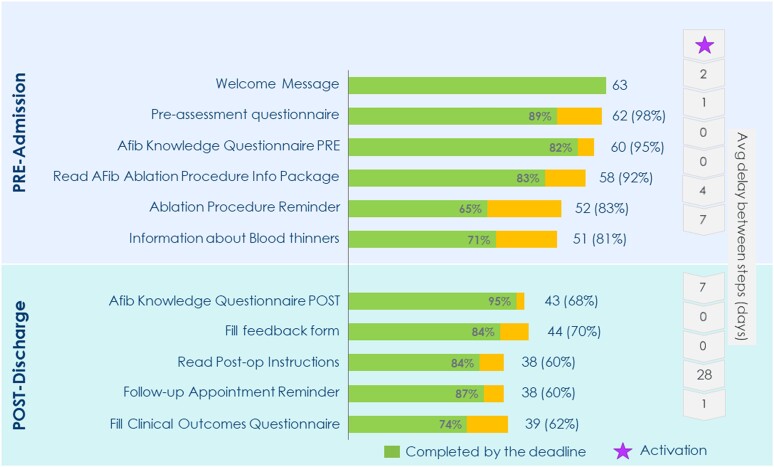
Patient compliance with the steps required by the treatment path and execution timings.

**Figure 3:**
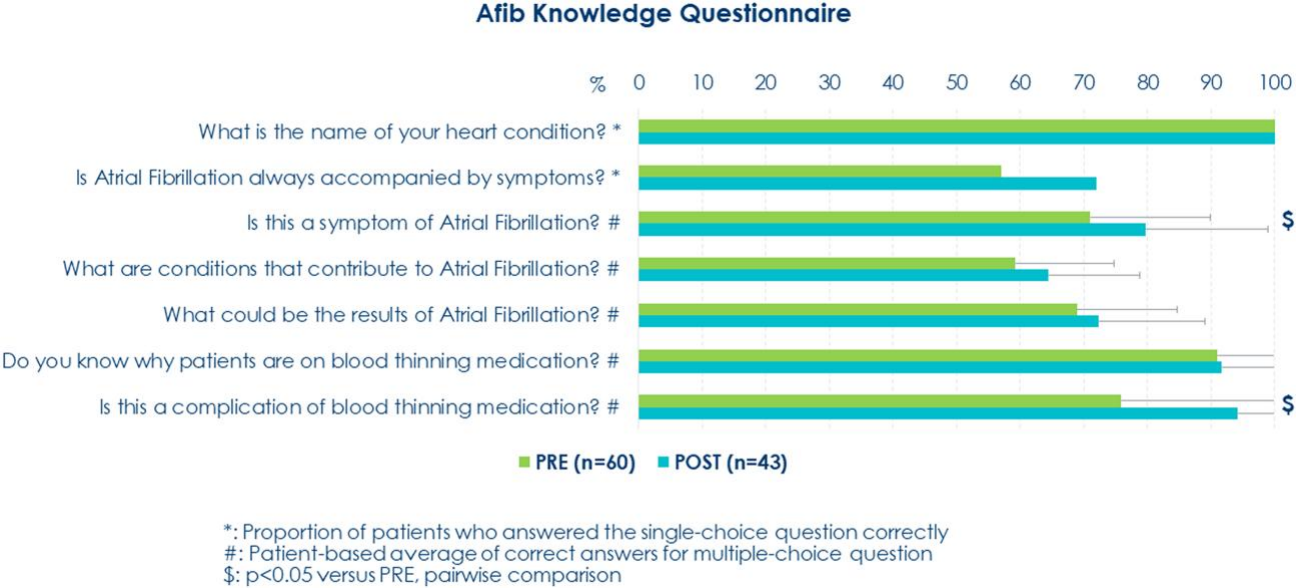
Survey questions on patient atrial fibrillation knowledge (PRE-admission: 60 respondents, POST-discharge: 43 respondents).

During the period spanning from September 2022 to April 2023, a total of 63 consecutive patients successfully installed the mobile application. All invited patients installed the application. The baseline characteristics of the population are detailed in *Table 1*. Four patients did not undergo the ablation procedure (three patients withdrew consent, and one found out she was pregnant). The remaining 59 patients underwent successful AF ablation, with the procedure involving the isolation of pulmonary veins using a point-by-point radiofrequency ablation catheter technique^7^ or pulsed field ablation with irreversible electroporation.^8^ No complications were reported during the procedures, and the post-operative period transpired without any noteworthy events for all patients. The adherence of patients to the prescribed steps within the treatment pathway, as well as their compliance with the specified execution timings, is depicted in *Figure 2*. Sixty-two (98%) patients actively engaged in at least one task within the pathway, with 81% of these individuals successfully completing all the tasks designated for the pre-operative phase. The average duration from enrolment to hospital admission was recorded at 14 days. Following the ablation procedure, the post-ablation pathway encompassed an average period of 36 days, and 62% of patients fulfilled all the requisite tasks. Overall, the planned actions were largely executed within the expected timelines. Survey responses regarding operator experiences with the Ablation Solution are reported in *Table 2*. The majority of operators (cardiac electrophysiologists and arrhythmia nurse specialists) found the system to be an effective tool for enhancing patient connectivity and optimizing the patient flow. However, they perceived fewer advantages in terms of reducing follow-up time and streamlining information collection. Overall, the platform was judged easy to use, complete, and met operators’ expectations. Additionally, operators recommended implementing additional features to enhance the application. In terms of patient information materials, they suggested the inclusion of descriptive procedure videos alongside a section addressing frequently asked questions. Furthermore, they expressed a preference for remote consent collection and patient self-registration to streamline the enrolment process and improve efficiency. Survey responses gauging patient experiences with the mobile application are documented in *Table 3*. The level of patient satisfaction was consistently high, with the application being appraised for its user-friendliness and the clarity of the information it provided. Notably, the application was independently installed by the patient in 91% of cases, with the caregiver or the patient’s doctor handling installation in the remaining instances. An analysis of questionnaire responses, pertaining to AF knowledge furnished to patients at the time of enrolment and post-hospital discharge, is presented in *Figure 3*. It was evident that patients had incomplete initial knowledge about the disease and its treatment. However, after completing the pathway and utilizing the informational resources available within the application, their knowledge exhibited noticeable improvement, particularly concerning the recognition of symptoms and anticoagulation therapy-related complications.

**Table 1:** Demographics and baseline clinical parameters

Parameter	*n* = 63
Male gender, *n* (%)	43 (68%)
Age, years	63 ± 12
History of atrial fibrillation, *n* (%)	
Paroxysmal	37 (59%)
Persistent	26 (41%)
Coronary artery disease, *n* (%)	8 (13%)
Heart failure, *n* (%)	1 (2%)
Hypertension, *n* (%)	30 (48%)
Diabetes mellitus, *n* (%)	5 (8%)
COPD, *n* (%)	0 (0%)
Chronic kidney disease, *n* (%)	3 (5%)
Peripheral arterial disease, *n* (%)	3 (5%)
LV ejection fraction < 50%, *n* (%)	3 (5%)
Prior stroke, *n* (%)	2 (3%)
CHA2DS2-VASc score (score ≥ 2)	32 (51%)
Ablation procedure, *n* (%)	59 (94%)
Point-by-point radiofrequency, *n* (%)	12 (20%)
Pulsed field ablation, *n* (%)	47 (80%)

COPD, chronic obstructive pulmonary disease; LV, left ventricular.

**Table 2:** Survey questions on operator experience with the Ablation Solution (seven respondents)

	Strongly disagree	Disagree	Neutral	Agree	Strongly agree
Factors that allowed the successful implementation of the Ablation Solution					
Support for carepath customization	0 (0%)	0 (0%)	1 (14%)	2 (29%)	4 (57%)
Project management for deployment	0 (0%)	0 (0%)	1 (14%)	4 (57%)	2 (29%)
Materials to support patient education and adoption	0 (0%)	0 (0%)	0 (0%)	3 (43%)	4 (57%)
The Ablation Solution allowed to					
Improve connectivity with patients	1 (14%)	0 (0%)	2 (29%)	0 (0%)	4 (57%)
Reduce the time needed to follow up with patients	0 (0%)	4 (57%)	3 (43%)	0 (0%)	0 (0%)
Collect patient information	0 (0%)	3 (43%)	3 (43%)	0 (0%)	1 (14%)
Streamline patient flow	0 (0%)	0 (0%)	1 (14%)	4 (57%)	2 (29%)
Reinforce the brand image of the hospital	0 (0%)	0 (0%)	2 (29%)	4 (57%)	1 (14%)
The Ablation Solution					
Was easy to use	0 (0%)	0 (0%)	2 (29%)	2 (29%)	3 (43%)
Offered the required functionalities to facilitate its use in clinical routine	0 (0%)	0 (0%)	0 (0%)	3 (43%)	4 (57%)
I would recommend it to another hospital	0 (0%)	0 (0%)	0 (0%)	2 (29%)	5 (71%)

**Table 3:** Survey questions on patient experience (44 respondents)

	Strongly disagree	Disagree	Neutral	Agree	Strongly agree
Application					
Was easy to use	0 (0)	0 (0)	1 (2)	31 (71)	12 (27)
Provided clear answers	1 (2)	0 (0)	5 (11)	24 (55)	14 (32)
Improved the level of satisfaction with the care at the hospital	0 (0)	0 (0)	3 (7)	28 (64)	13 (29)
I would recommend it to another patient	0 (0)	0 (0)	0 (0)	32 (73)	12 (27)
Application used by					
Patient alone	40 (91)				
Patient with caregiver	3 (7)				
Patient’s doctor	1 (2)				

## Discussion

We have described the implementation of a solution that combines a customized treatment pathway with a patient mobile application, aimed at optimizing the management of patients referred to our centre for AF catheter ablation. The solution has been employed by the patients, effectively guiding them along their care pathway and enhancing their understanding of the disease and available therapies. The adoption of the system was widespread, particularly for pre-procedure tasks. Adherence was commendable, and patients reported a positive user experience, even though most used the system independently, without assistance from caregivers. These patients were not predominantly elderly and may have been in better overall health than those with other conditions for which similar aids have been proposed in the literature. It’s worth mentioning that for other chronic diseases, such as diabetes and heart failure, disease management programmes have shown not only significant improvements in patient knowledge but also a positive impact on readmission and mortality.^7,9–13^ The implementation of guideline-recommended management to individual AF patients aims to enhance patient outcomes and reduce healthcare costs.^14–16^ Integrated AF management has the potential to promote adherence to these guidelines, which is modest worldwide.^17–22^ Various educational interventions,^23–32^ based on guideline-provided recommendations and tailored to address specific knowledge gaps among AF patients, can facilitate the implementation of guideline-based AF management to improve patient outcomes. Clinical decision-support systems, intelligent tools that digitize and provide evidence-based guidelines, clinical pathways, and algorithms for personalized, timely, and evidence-based treatment, have been used to enhance patient education, improve communication between patients and healthcare professionals, and encourage active patient involvement.^23,33–36^ The mobile AF (mAF) App Trial^23^ incorporated clinical decision-support tools, educational materials, and patient involvement strategies with self-care protocols and follow-up. Results showed significant improvements in knowledge, drug adherence, quality of life, and anticoagulation satisfaction. Similarly, the pilot study on Mobile Applications for Seniors to enhance Safe anticoagulation therapy (MASS)^35^ demonstrated improved knowledge of oral anticoagulation therapy among older adults. The Characterizing Atrial fibrillation by Translating its Causes into Health Modifiers in the Elderly (CATCH ME) Consortium^34^ developed mobile applications to engage patients, optimize therapy, and enhance outcomes in AF, illustrating the value of integrating digital technology into clinical practice. However, studies on the effectiveness of integrated AF management have yielded mixed results,^37–39^ and further research is needed to identify cost-effective intervention types that could more effectively enhance patient clinical outcomes, medication adherence, and quality of life.

In our experience, we observed that patients generally adhered well to the deadlines for various tasks, which is a positive outcome and certainly facilitates patient management throughout their treatment journey. Moreover, healthcare providers agreed that the system can offer an advantage in improving the connection with patients and streamlining patient flow. However, the system was not perceived as particularly advantageous for data collection and reducing follow-up time, possibly because some tasks still required in-person attention. The process might be further improved, or the benefits could become more tangible with longer post-procedure follow-up to evaluate arrhythmia recurrences and maintain a stronger patient connection. Post-procedure compliance was not absolute within our population, and this could be improved by providing more reminders to patients or by being more selective in patient enrolment in the care pathway. Nevertheless, according to healthcare providers, the system offered the necessary functionalities to facilitate its integration into clinical routine.

In our project, we prioritized patient education in terms of information and instruction, as these components are critical in the management of AF. Many studies have primarily focused on knowledge related to anticoagulation therapy in AF patients or associated cardiovascular risks.^40–46^ Overall, patients’ knowledge in these areas is lacking, with a low proportion of patients being aware of their cardiac condition and the reasons for initiating anticoagulation therapy.^40,41^ Understanding the nature and consequences of AF and appropriate therapy is essential for AF self-management.^5,8^ A previous study demonstrated the need for specific education programmes and their contribution to cardiovascular morbidity and mortality in AF patients.^45^ AF catheter ablation is a complex procedure that may be associated with a range of specific post-procedural complications. Although mostly rare, potentially serious complications may initially present with non-specific symptoms and signs. International recommendations emphasize the importance of fully informing patients about the clinical signs and symptoms of ablation-related complications that may occur after hospital discharge and the significance of assessing procedural success and correlating symptom status with rhythm.^1^ Moreover, although clinical practice regarding antiarrhythmic medication and anticoagulation therapy after ablation varies, there is a need for careful patient follow-up, adherence to indicated treatments, and monitoring of stroke risk factors and rhythm status. In these aspects, the system proved to be effective. We confirmed that, although our patients already had a diagnosis of AF and were referred for an ablative procedure (many with a relatively long history of the disease and some in persistent AF), they did not possess sufficient knowledge of the disease and treatments. Primarily, the system improved patients’ knowledge of symptoms and treatments through informational materials that patients could access independently. Furthermore, the system is entirely customizable, offering the potential for further improvements based on specific needs.

### Limitations

Our findings should be considered in light of potential limitations. This project was conducted at a single centre within a relatively short time frame in a non-randomized fashion. In addition, to test the knowledge of patients with AF, we decided to use a subset of questions from a validated questionnaire^3^ to make data collection through the mobile application less burdensome for the patients.

### Future perspectives

To establish the system’s effectiveness more conclusively, further research involving larger populations and the assessment of well-defined, clinically relevant endpoints (e.g. clinical events and arrhythmia recurrences), as well as validated and psychometrically tested instruments (e.g. quality of life and knowledge questionnaire), is necessary and currently being planned. In addition, evidence is necessary regarding efficiency for clinicians and support staff (e.g. total workload, number of patient contacts, reduction in errors, appointment cancellations, or scheduling adjustments).

## Conclusions

In conclusion, our findings showed the successful implementation of a comprehensive solution integrating a tailored treatment pathway and a patient mobile application for optimizing the management of individuals referred to our centre for AF catheter ablation. The adopted solution demonstrated widespread use among patients, effectively guiding them through their care journey, improving their understanding of the disease and available treatments.

## Supplementary Material

ztae041_Supplementary_Data

## Data Availability

The data that support the findings of this study are available on request from the corresponding author.
