# Supplementary material for: Streamlining atrial fibrillation ablation management using a digitization solution
Source: Eur Heart J Digit Health. 2024 May 23;5(4):483–90. doi: 10.1093/ehjdh/ztae041 (PMC11284009; doi:10.1093/ehjdh/ztae041)
Supplement: ztae041_Supplementary_Data [file ztae041_supplementary_data.docx]

**Supplemental Figure 1.** Example screenshots of the application (Messages, Questionnaires, Educational materials).


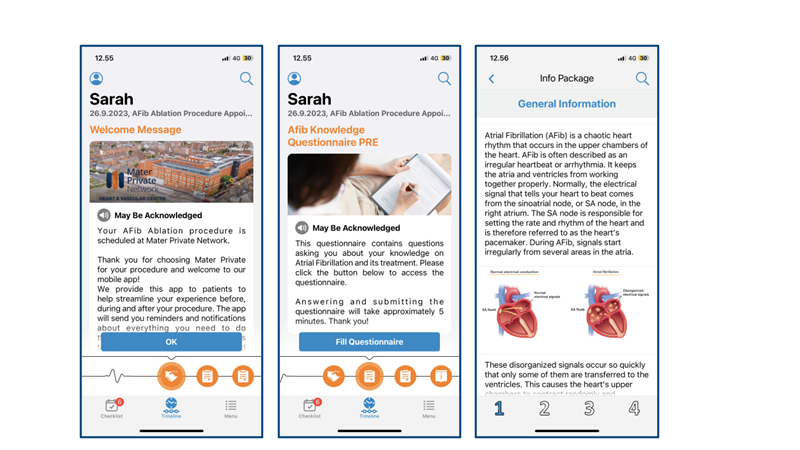
**Supplemental Figure 2.** Web-based dashboard for the center.


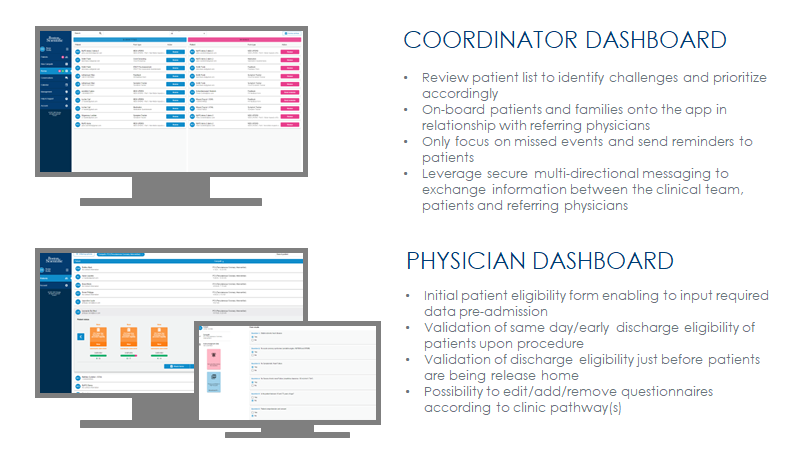


**Supplemental Table 1.** Patient data, patient reported outcomes and checklists collected using the platform.

| **Clinical Pre-assessment Questionnaire (Filled-in by clinical team)** | | |
| --- | --- | --- |
| **Clinical history** | | |
| **Medications** | | |
| **COVID-19 screening** | | |
| **AFib Ablation Same Day Discharge checklist (Filled-in by clinical team)** | | |
| **Pre-procedure eligibility** | **Clinical criteria** | Stable anticoagulation? |
|  |  | Absence of bleeding history? |
|  |  | No systolic heart failure? |
|  |  | No history of pulmonary disease? |
|  |  | No interventional procedures within 60 days of ablation? |
|  |  | BMI < 35 (recommended)? |
|  |  | Acceptable CHA2DS2-VASc stroke risk (typically ≤3)? |
|  |  | Results of Frailty test (Clinical Frailty Scale ≤3)? |
|  | **Socio-economic criteria** | Is the patient between 18 and 75 years of age? |
|  |  | Patient comprehension and consent to SDD? |
|  |  | Patient's home less than 60 min from hospital. Patient has reliable transportation. |
|  |  | Care-giver at home after discharge? |
|  |  | Working phone at home? |
| **Peri-procedure eligibility** |  | Procedure was completed before 2pm? |
|  |  | No in-lab complications? |
|  |  | No bleeding events? |
|  |  | No side effects of sedation/anesthesia? |
|  |  | Duration of procedure <2 h (general anesthesia)/<90 mins (conscious sedation)? |
|  |  | No neurological symptoms? |
| **Post-procedure eligibility** |  | Procedure occurred without complications? |
|  |  | Confirmation was obtained from the operator to proceed with SDD? |
|  |  | Purse string suture removed? |
|  |  | Stable hemodynamics? |
|  |  | No evidence of groin or respiratory complications? |
|  |  | Patient able to tolerate liquids/food? |
|  |  | Patient able to ambulate? |
|  |  | Glomerular Filtration Rate; <30ml/min/1.73m²? |
|  |  | O₂ Saturation >95% on room air? |
|  |  | Systolic BP >90; HR <100? |
|  |  | No pericardial effusion on TTE prior discharge? |
|  |  | No complications during post-procedure observation time? |
|  |  | No bleeding? |
|  |  | Patient had urine output in the recovery area? |
| **Clinical Outcomes Questionnaire (Filled-in by patients)** | | |
| I am satisfied with the length of my recovery. | | |
| I did not experience any late complications. | | |
| I did not experience any new cardiac symptoms. | | |
| I did not experience any issues regarding my medication change. | | |
| I am satisfied overall with my AFib ablation procedure. | | |
| I was able to return to my previous level of activity/lifestyle. | | |
| My quality of life has improved after the procedure. | | |
| **EQ-5D-3L Health Questionnaire (Filled-in by patients)** | | |

**Supplemental Table 2.** Atrial fibrillation knowledge questionnaire.

|  |  | **Select correct answer(s):** [Correct answers in bold] |
| --- | --- | --- |
| **Atrial Fibrillation**  **knowledge** | **What is the name of your heart condition?** | - **Atrial Fibrillation** - Ectopic heartbeats - Ventricular fibrillation - WPW syndrome - Ischemic heart disease |
|  | **Is Atrial Fibrillation always accompanied by symptoms?** | - Yes - **No** |
|  | **Is this a symptom of Atrial Fibrillation?** | - **Irregular Heartbeat** - **Breathlessness** - **Chest Discomfort** - **Light-headedness** - **Decreased Exercise Ability** - **Low energy levels** - Loss of consciousness - Speech disorders - Loss of strength in the limbs - **Atrial fibrillation can present without symptoms** |
|  | **What are conditions that contribute to Atrial Fibrillation?** | - **Age** - **Overweight** - **High blood pressure** - **Diabetes mellitus** - **Chronic lung disease** - **Sleep apnea** - High cholesterol - History of heart attacks in the family - **Heart valve problems** - Recurrent infections - Moderate intensity exercise |
|  | **What could be the results of Atrial Fibrillation?** | - **Reduced quality of life due to symptoms** - **Stroke** - **Heart Failure** - **Heart Attack** - Lung disease - **Heart valve problems** - **Dementia** - Cancer - Eating disorders |
| **Treatment knowledge** | **Do you know why patients are on blood thinning medication?** | - **To avoid blood clots which can lead to stroke** - To prevent AFib permanently - To control the heart rate - To prevent heart attacks - I don’t know |
|  | **Is this a complication of blood thinning medication?** | - **Bruising** - **Bleeding** |
